# Supplementary material for: Content-rich biological network constructed by mining PubMed abstracts
Source: BMC Bioinformatics. 2004 Oct 8;5:147. doi: 10.1186/1471-2105-5-147 (PMC528731; doi:10.1186/1471-2105-5-147)
Supplement: Additional File 2 — The original results of the above study (non-essential files are deleted to keep the file size under the limit set by BMC bioinformatics). [file 1471-2105-5-147-S2.bz2 › chilibotAdditionalFile2/dip20/617ID10493800E11001/html/CALM1_ATP2B4.html]

 


 **CALM1** and **ATP2B4** 
  
Found 13 abstracts in PubMed, retrieved 13.  
 

 What does Google say? 
 PDF only 
| .edu only 

---

**Interactive relationship** (e.g. stimulation, inhibition, etc)

**Neutral relationship**- The affinity of PMCA1 for calmodulin  [ **CALM1** ]  was similar to that of PMCA4  [ **ATP2B4** ] , the other ubiquitous PMCA isoform.  Ref: 12851406 J Biol Chem< MedlineTA>J Biol Chem,
- This alternately spliced form of the PMCA4  [ **ATP2B4** ]  mRNA contained an exon inserted at the splice junction immediately following the sequence encoding the calmodulin  [ **CALM1** ]  binding domain.  Ref: 1531651 J Biol Chem, 1992

**Non-interactive relationship** (e.g. studied together, co-existance, homology, etc.)

- By contrast, Ca2 independent activity of the calmodulin  [ **CALM1** ]  dependent protein kinase II CaMK II increased eightfold as MOVAS progressed from G0 to G1 S, and treatment with an inhibitor of CaMK II KN 93 or transduction of a c Myb neutralizing antibody significantly alleviated the G1 S associated repression of PMCA4  [ **ATP2B4** ] .  Ref: 12660151 Am J Physiol Cell Physiol, 2003
- The Ca 2 dependent ATPase activity of the PMCA2 pumps had a 5 10 fold higher affinity for calmodulin  [ **CALM1** ]  than the PMCA4  [ **ATP2B4** ]  pump expressed in the same system.  Ref: 7929331 J Biol Chem, 1994
- At variance with the corresponding truncated variant of PMCA4  [ **ATP2B4** ] , which had been studied previously PMCA3CII 3a had very high calmodulin  [ **CALM1** ]  affinity.  Ref: 12716903 J Biol Chem< MedlineTA>J Biol Chem, 1994
- The purified PMCA4CII had a 20 fold lower affinity for calmodulin  [ **CALM1** ]  than the PMCA4CI, the PMCA4  [ **ATP2B4** ]  isoform of the erythrocytes membranes, but had a higher activity in the absence of calmodulin  [ **CALM1** ] .  Ref: 8672497 Biochemistry, 1996
